# Design of a novel stimulation system with time-varying paradigms for investigating new modes of high frequency stimulation in brain

**DOI:** 10.1186/s12938-018-0523-3

**Published:** 2018-06-22

**Authors:** Ziyan Cai, Zhouyan Feng, Hanhan Hu, Na Hu, Xuefeng Wei

**Affiliations:** 1 Key Laboratory of Biomedical Engineering of Education Ministry, College of Biomedical Engineering & Instrument Science, Zhejiang University, Hangzhou, 310027 Zhejiang China; 2 Department of Biomedical Engineering, The College of New Jersey, Ewing, NJ 08628 USA

**Keywords:** High frequency stimulation, LabVIEW software, Time-varying intensity, Time-varying frequency, Axonal block

## Abstract

**Background:**

Deep brain stimulation (DBS) has shown wide clinical applications for treating various disorders of central nervous system. High frequency stimulation (HFS) of pulses with a constant intensity and a constant frequency is typically used in DBS. However, new stimulation paradigms with time-varying parameters provide a prospective direction for DBS developments. To meet the research demands for time-varying stimulations, we designed a new stimulation system with a technique of LabVIEW-based virtual instrument.

**Methods:**

The system included a LabVIEW program, a NI data acquisition card, and an analog stimulus isolator. The output waveforms of the system were measured to verify the time-varying parameters. Preliminary animal experiments were run by delivering the HFS sequences with time-varying parameters to the hippocampal CA1 region of anesthetized rats.

**Results:**

Verification results showed that the stimulation system was able to generate pulse sequences with ramped intensity and hyperbolic frequency accurately. Application of the time-varying HFS sequences to the axons of pyramidal cells in the hippocampal CA1 region resulted in neuronal responses different from those induced by HFS with constant parameters. The results indicated important modulations of time-varying stimulations to the neuronal activity that could prevent the stimulation from inducing over-synchronized firing of population neurons.

**Conclusions:**

The stimulation system provides a useful technique for investigating diverse stimulation paradigms for the development of new DBS treatments.

## Background

Deep brain stimulation (DBS) has become an effective treatment for movement disorders that occur in Parkinson’s disease and essential tremors [1, 2]. The advantages of DBS over pharmacological treatments include: good spatial and temporal specificity, reversible effects, fewer side effects, and feasible adjustments of stimulation parameters to maximize efficacy [3]. Thus, in recent years, DBS has been investigated intensively as a promising treatment for a variety of other neurological and psychiatric diseases, such as refractory epilepsy [4], chronic pain [5], and resistant depression [6]. Presently, regular DBS utilizes sequences of high frequency stimulation (HFS, over 100 Hz) of narrow pulses with constant intensities and constant frequencies. To meet the expanding demands of DBS treatments for different diseases in various brain regions, diverse stimulation paradigms with time-varying parameters are worth to explore [7, 8]. For example, previous studies have shown that synchronous firing of population neurons (i.e., epileptiform activity) can be induced at the initial phases of HFS with fixed parameters [9, 10], which may cause transient side effects in clinic therapy of DBS [11]. New stimulation paradigms need to be developed to avoid the “epileptiform activity” for a safer use of DBS.

However, most commercial stimulators do not allow generating a pulse sequence with arbitrary time-varying parameters. They can only deliver pulses at a fixed frequency and a fixed intensity from the onset of a stimulation session to its end [12, 13]. Although an analog signal generator can output arbitrary waveforms to control a stimulator, it lacks a friendly interface to program output waveforms on-line. Whereas, the software LabVIEW, a tool for virtual instrument (VI), can be applied to control stimulator hardware via graphical user interfaces (GUI) [14, 15]. Therefore, in this study, we developed a stimulation system by utilizing the LabVIEW technique of virtual instrument to overwrite a data acquisition (DAQ) card that controlled a commercial stimulator.

Stimulation paradigms with time-varying intensities and time-varying frequencies were generated and delivered into rat hippocampal region to test the functionality of the stimulation system, as well as the feasibility of modulating neuronal activity by time-varying stimulations in in vivo experiments. As a demonstration, we designed pulse sequences with a ramped-intensity phase and an attenuating-frequency phase to eliminate synchronized firing of population neurons in the initial period of HFS. The amplitude of HFS-evoked population spikes was measured as an electrophysiological index to evaluate the changes of synchronicity of neuronal firing [16]. The design may facilitate the investigation of novel stimulation paradigms for advancing the applications of DBS.

## Methods

### Configuration of stimulation system

**Fig. 1:**
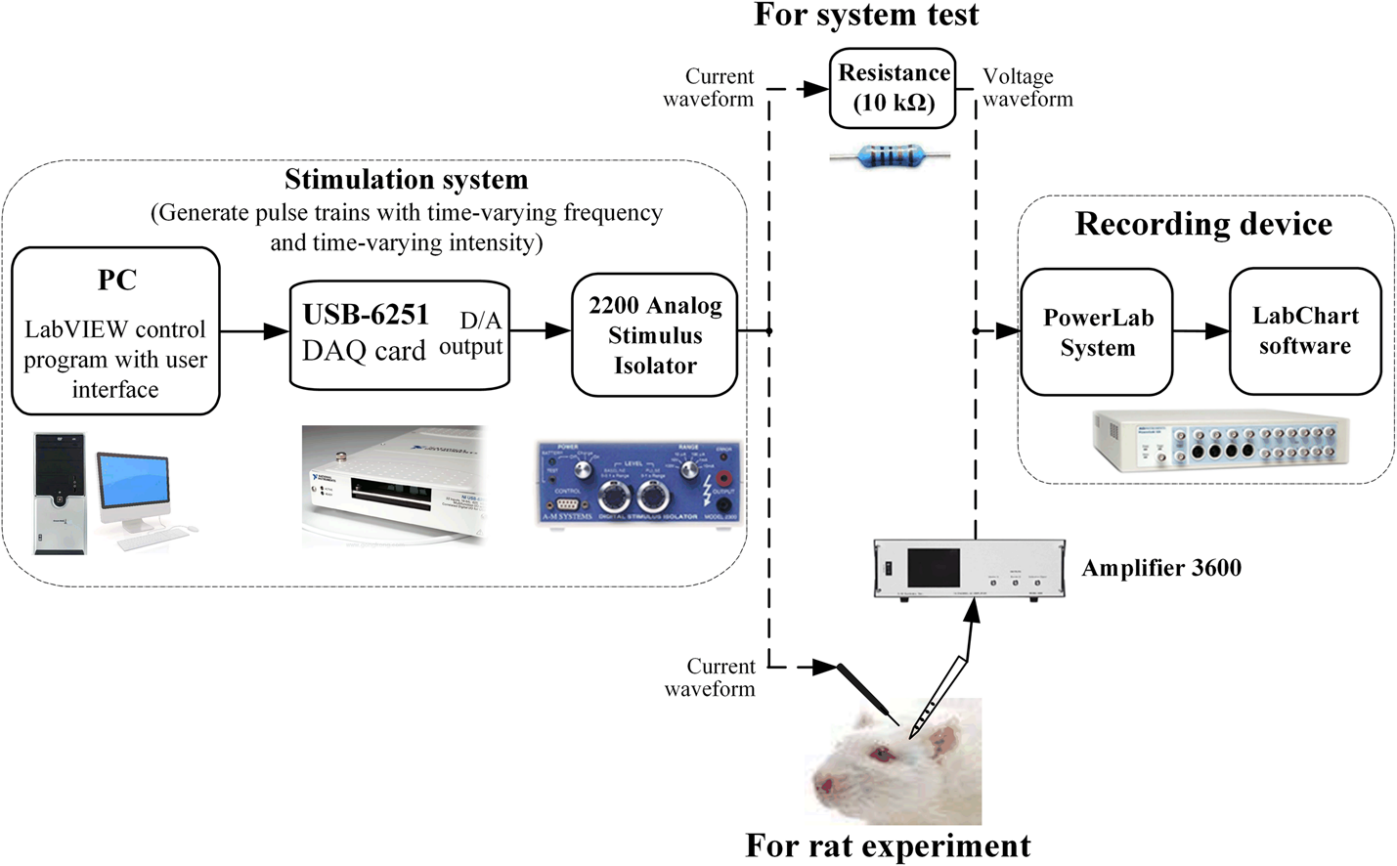
Schematic diagram of the stimulation system (left), test connections or experiment connections (middle), and the recording device (right)

To generate stimulus pulses with time-varying frequency and time-varying intensity, we used a personal computer (PC), a USB-6251 DAQ card (National Instruments), and a 2200 analog stimulus isolator (A-M Systems Inc.). The PC ran a custom-made LabVIEW program to control one of the D/A converters in the DAQ card to generate desired voltage waveforms of stimulation pulses. The voltage waveforms were then converted into a sequence of current pulses by the 2200 stimulus isolator (Fig. 1 *left*). The sequence of current pulses would be finally delivered into a rat brain through a stimulation electrode.

To verify the output sequence of pulses generated by the stimulation system, we applied the output current signals on a 10 kΩ resistor and measured the voltage signals across the resistor by using a ML880 PowerLab DAQ system with its software LabChart 7 (ADInstruments Inc.) (Fig. 1, *middle* and *right*).

### Design of LabVIEW control program and the stimulation waveforms

LabVIEW control program of the stimulation system includes modules of initialization, user interface, settings of stimulation parameters, generation of pulse sequences, and output of stimulation waveforms. The key function of the LabVIEW program is to generate sequences of biphasic pulses with time-varying parameters. The details of the design are described below.

#### Generation of basic biphasic pulses

**Fig. 2:**
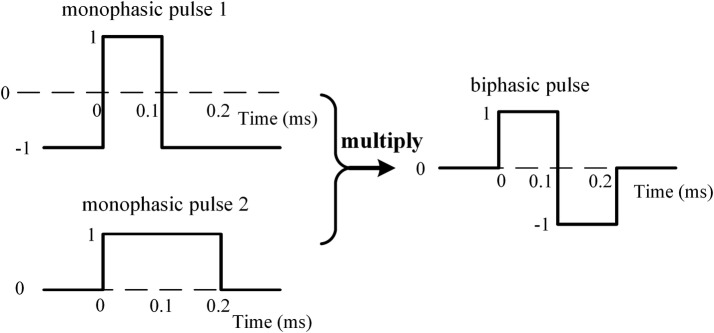
Biphasic pulse generated by a LabVIEW sub-VI that multiplied two monophasic pulses

A symmetric biphasic pulse was designed as basic stimulation waveform to meet the charge-balance requirement of neural electrical stimulation [17]. The width of each phase of the biphasic pulse was 0.1 ms. Because no function or VI in LabVIEW allows generating a biphasic pulse directly, the built-in VI “signal generator by duration” was used to generate two sequences of monophasic pulses. Then, the two sequences were multiplied into a biphasic waveform sequence (Fig. 2). Thus, a sub-VI “biphasic pulse” was coded to generate a basic pulse sequence with a constant frequency and one unit of intensity. Multiplying the basic sequence by an intensity coefficient could generate pulses with any desired intensity. Although the original output of biphasic pulses was anodic phase first, switching the two output cables of the 2200 stimulus isolator would reverse the final waveform and generate pulses with cathodic phase first.

#### Generation of a stimulation sequence with ramped intensity

To generate pulses with linearly increasing intensity, the LabVIEW VI “signal generator by duration” was used again with “increasing ramp” option and settings of a start value and a ramped value to generate a ramped line. Multiplying the ramped line with the previous sub-VI “biphasic pulse” resulted in a sequence of biphasic pulses with ramped intensity.

#### Generation of a stimulation sequence with time-varying frequency

Three parameters were given for a sequence with time-varying frequency: stimulation duration *T*, initial frequency *f*_1_ and terminal frequency *f*_*N *− 1_. The interval between two neighboring pulses was denoted as $t_{i} = 1/f_{i};\,( 1\le i \le N - 1),$ where *N* is the total number of pulses for the stimulation sequence. Then, a pulse sequence with time-varying frequency was obtained by linearly changing the intervals *t*_*i*_. The values of *N* and *t*_*i*_ were determined as following:

1 $$T = \frac{{(t_{1} + t_{N - 1} )(N - 1)}}{2}$$

thus

2 $$N = \frac{2T}{{\left( {t_{1} + \left. {t_{N - 1} } \right)} \right.}} + 1 = \frac{2T}{{\left( {\frac{1}{f_{1}} + \left. {\frac{1}{f_{N - 1}}} \right)} \right.}} + 1$$

and

3 $$t_{i} = \frac{{t_{N - 1} - t_{1} }}{N - 2}(i - 1) + t_{1} = \frac{{\frac{1}{{f_{N - 1} }} - \frac{1}{{f_{1} }}}}{N - 2}(i - 1) + \frac{1}{{f_{1} }}.$$

A LabVIEW sub-VI was coded for the calculation of Eqs. (2) and (3). The obtained values of *N* and *t*_*i*_ joined with the basic sub-VI “biphasic pulse” to generate a desired stimulation sequence with time-varying frequency.

In the present study, we tested a pulse sequence with an elevated pulse-frequency (400 Hz) initially that was then attenuated to 100 Hz. We call this type of stimulation attenuating-frequency HFS below.

#### Output of stimulation waveforms

A built-in function “NI-DAQmx” of LabVIEW was used to drive the D/A converter of the USB-6251 DAQ card and then to control the 2200 stimulus isolator to generate desired sequences of stimulation pulses. The “NI-DAQmx” function was set as a single-channel output of analog voltage via one of the D/A converters with a sampling rate of 20 kHz to ensure adequate resolution for the 0.1 ms pulse width. The sampling rate could be changed on the GUI panel. The final output sequence was built by using the LabVIEW VI “Append Signals Express” to sequentially combine several pieces of pulse sequences with required parameters to form a compound stimulation sequence.

### In vivo animal experiment and data recording

All procedures involving animal care and experiments conformed to the Guide for the Care and Use of Laboratory Animals (China Ministry of Health). The surgical procedure and data collection methods were similar to previous reports [10, 18]. Briefly, adult Sprague-Dawley rats (250–400 g) were anesthetized with urethane (1.25 g/kg, i.p.) and placed in a stereotaxic apparatus. Electrodes were inserted into the hippocampal CA1 region through partially opened skull. A stimulation electrode, concentric bipolar electrode (Model CBBSC75, FHC), was positioned in the alveus, the efferent fiber tracts of hippocampal CA1 region, for antidromically exciting the CA1 pyramidal cells. A recording electrode (16-channel array, Model Poly2, NeuroNexus Technology) was positioned in the CA1 pyramidal cell layer, upstream of the stimulating site, to collect the antidromically-evoked neuronal responses.

The electrophysiological potentials collected by the recording electrode array were amplified by a 16-channel extracellular amplifier (Model 3600, A-M System Inc.) and were then sampled by a PowerLab data acquisition system at a sampling rate of 20 kHz/channel.

## Results

### Verification tests of the stimulation system

**Fig. 3:**
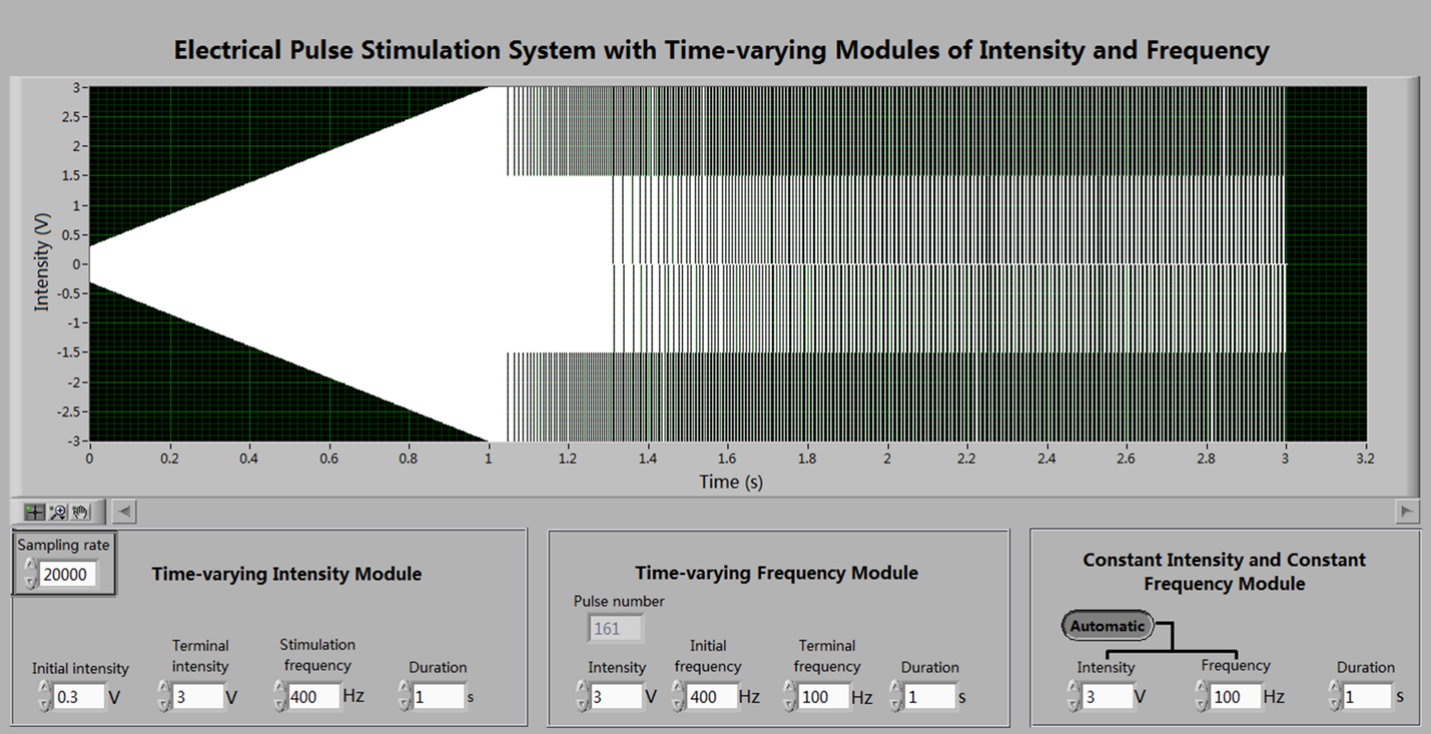
User interface of the stimulation system

The user interface of the stimulation system includes two panels (Fig. 3). The top panel displays the output of stimulation waveform. The bottom panel is for parameter settings of three modules of stimulation sub-sequences: time-varying intensity, time-varying frequency, and constant intensity and frequency. The parameters of time-varying intensity include: initial intensity, terminal intensity, stimulation frequency and duration. Similarly, the parameters of time-varying frequency include: intensity, initial frequency, terminal frequency and duration. In addition, the pulse number is calculated by the system automatically and displayed as a reference. The parameters of constant intensity and constant frequency include: intensity, frequency and duration. Additionally, an option button “automatic” is designed for automatically succeeding the values of intensity and frequency from a preceding sub-sequence. The entire stimulation sequence is the sequential outputs of the above three sub-sequences. Setting a duration as “0” allows omitting a particular sub-sequence. Finally, the setting of sampling rate of output stimulation signals is on the leftmost corner with a default value of 20 kHz.

The voltage output of the system was converted into current pulses by a 2200 stimulus isolator that was set at an output mode 0.1 mA/V. Thus, a voltage setting of 0.3–3 V in the system is corresponding to a current intensity of 0.03–0.3 mA.

Test results showed adequate accuracies for all the parameters of the output pulses. For a test sequence with constant intensity 3 V, constant frequency 100 Hz and a duration 5 s (total 500 pulses), the percent differences between the setting values and measured values were within 1% for intensity, frequency and duration, and 2.7% for a pulse width setting of 0.1 ms (Table 1).

**Table 1:** Measured values of the stimulation parameters

Parameters	Settings	Measured values (mean ± standard deviation)	|Mean-Setting|/Setting (%)
Intensity (V)	3	3.0112 ± 0.0005	0.37
Frequency (Hz)	100	100.0010 ± 0.0388	0.001
Duration (s)	5	4.9982 ± 0.0038	0.036
Pulse width (ms)	0.1	0.1027 ± 0.0037	2.7

Number of pulse samples is 500

**Fig. 4:**
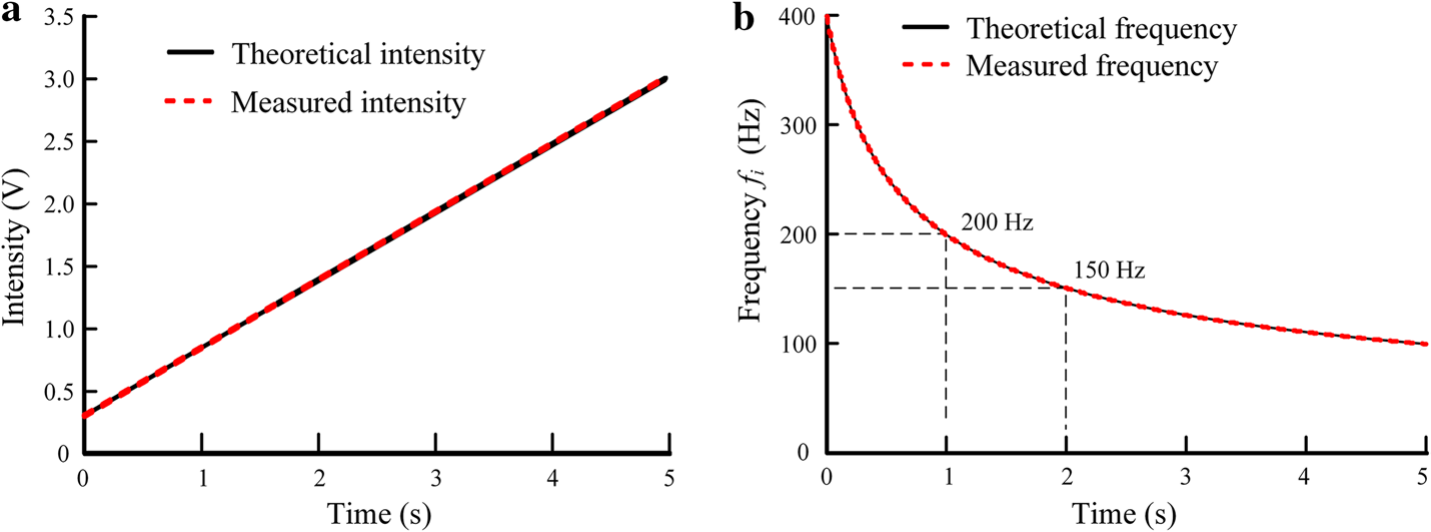
Test results for pulse sequences with time-varying intensity and with time-varying frequency. **a** Comparison between the theoretical intensity and measured intensity for a pulse sequence with time-varying intensity 0.3–3.0 V, pulse frequency 400 Hz and duration 5 s. **b** Comparison between the theoretical frequency and measured frequency for a pulse sequence with time-varying frequency 400–100 Hz, intensity 3 V and duration 5 s

The measured intensities of a 5 s pulse sequence with linearly-increasing intensity from 0.3 to 3.0 V overlapped with the theoretical intensity line (Fig. 4a). Similarly, the measured frequencies of a 5 s pulse sequence with attenuating frequency from 400 to 100 Hz also overlapped with the theoretical frequency curve calculated from Eq. (3) in “Generation of a stimulation sequence with time-varying frequency” section (Fig. 4b). In addition, the time-varying frequency designed by linearly increasing pulse intervals resulted in a hyperbolic decline with a sharp initial drop gradually flatting down to the final desired frequency. Such a design could shorten the period of higher frequencies thereby saving electrical power. According to the curve in Fig. 4b, ~ 2/3 (400–200 Hz) of the total frequency fall of 300 Hz (400–100 Hz) occurred in the first 1/5 duration, and ~ 5/6 frequency fall completed in the first 2/5 duration. Test results with other values of duration, intensity and frequency were in consistent with the results in Fig. 4.

### Validation of time-varying stimulations in rat hippocampus

**Fig. 5:**
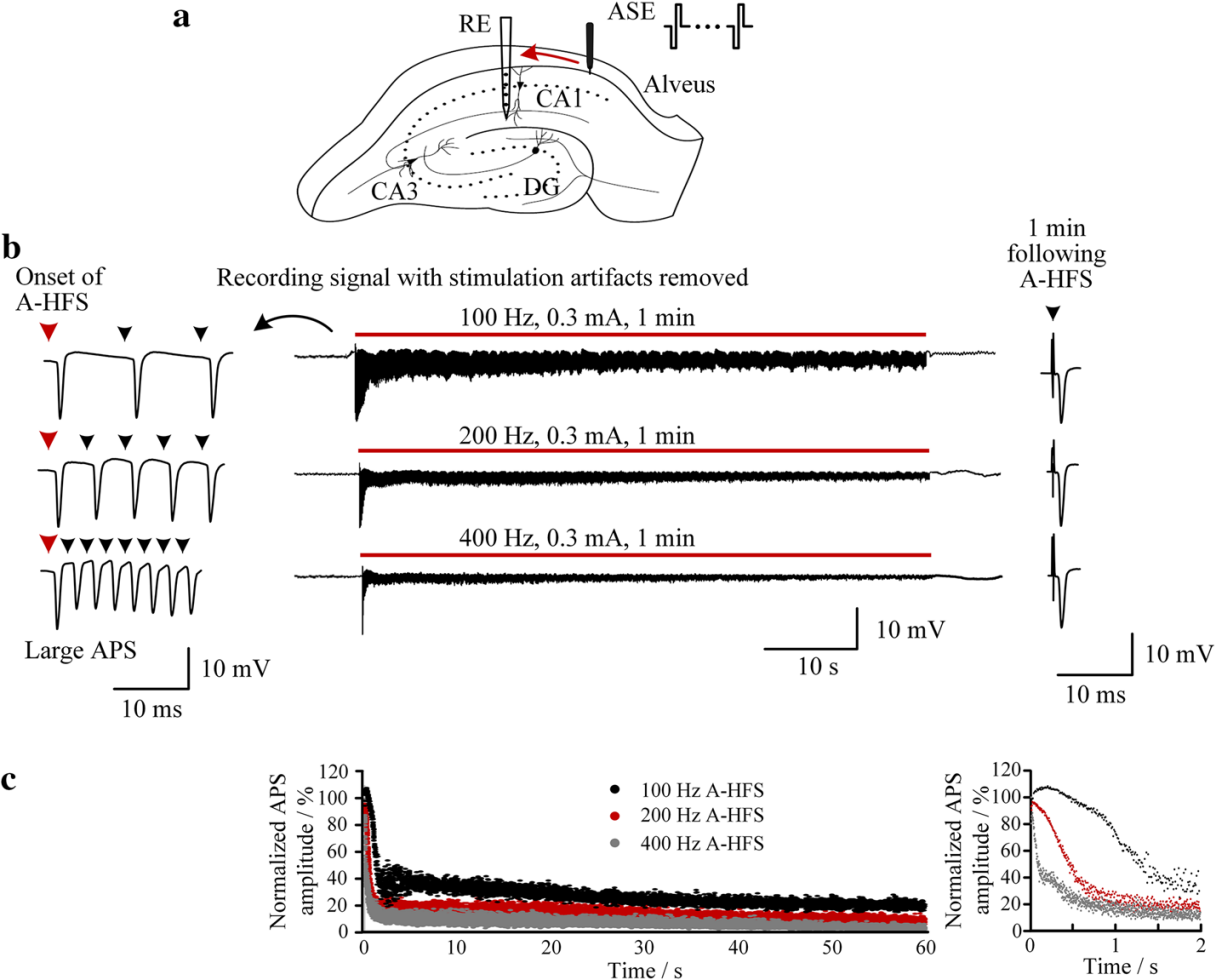
Neuronal responses to stimulation paradigms with various pulse frequencies. **a** Schematic diagram of a recording electrode (RE) in the CA1 pyramidal cell layer and an antidromic stimulation electrode (ASE) in the alveus of rat hippocampus. **b** Example of CAl neurons’ responses to 1-min A-HFS with constant parameters of an intensity 0.3 mA and a frequency 100, 200 and 400 Hz, respectively. Large APS events were always evoked by pulses at the initial phase of A-HFS with constant parameters. Red bars indicate the periods of A-HFS. **c** Scatter diagrams of the amplitudes of APS evoked by each pulse (normalized by the baseline APS evoked by a single pulse) during the whole 1-min A-HFS (*left*) and during the initial 2 s period (*right*)

The alveus of the hippocampus includes myelinated axon fibers arisen from the pyramidal cells in the hippocampal CA1 region (Fig. 5a). During extracellular stimulation of alveus fibers in vivo, axonal excitation may propagate antidromically and induce action potentials in the cell bodies of CA1 [19]. We used the amplitudes of the antidromically-evoked population spikes (APS) to evaluate the effects of antidromic high-frequency stimulation (A-HFS) with various paradigms on neuronal responses.

When an A-HFS sequence with certain constant parameters of intensity and frequency was applied to the alveus, the CA1 neurons responded by a transient phase before establishment of a steady phase. The transient phase was characterized by large APS events evoked by each pulse at the initial period of A-HFS. Then the amplitudes of evoked-APS gradually decreased to small values of steady phase (Fig. 5b). Consistent with previous studies [10, 18], the decrease of APS amplitude was more rapid with a higher stimulation frequency (Fig. 5c). However, even when the frequency was as high as 400 Hz, large APS still appeared at the initial phase of A-HFS (Fig. 5b, c). Nevertheless, A-HFS with a higher frequency induced a smaller number of large APS at the initial phase (Fig. 5c *right*), indicating a higher frequency could shorten the transient phase.

Large APS events are epileptiform activity that results from synchronous firing of a large population of neurons [20]. Thus, they should be avoided. Based on the neuronal responses to stimulations of different frequencies (Fig. 5), we hypothesize that using a smaller intensity and a higher frequency at the initial phase of stimulation could eliminate large APS. Therefore, we tested the hypothesis with the newly-designed stimulation system.

**Fig. 6:**
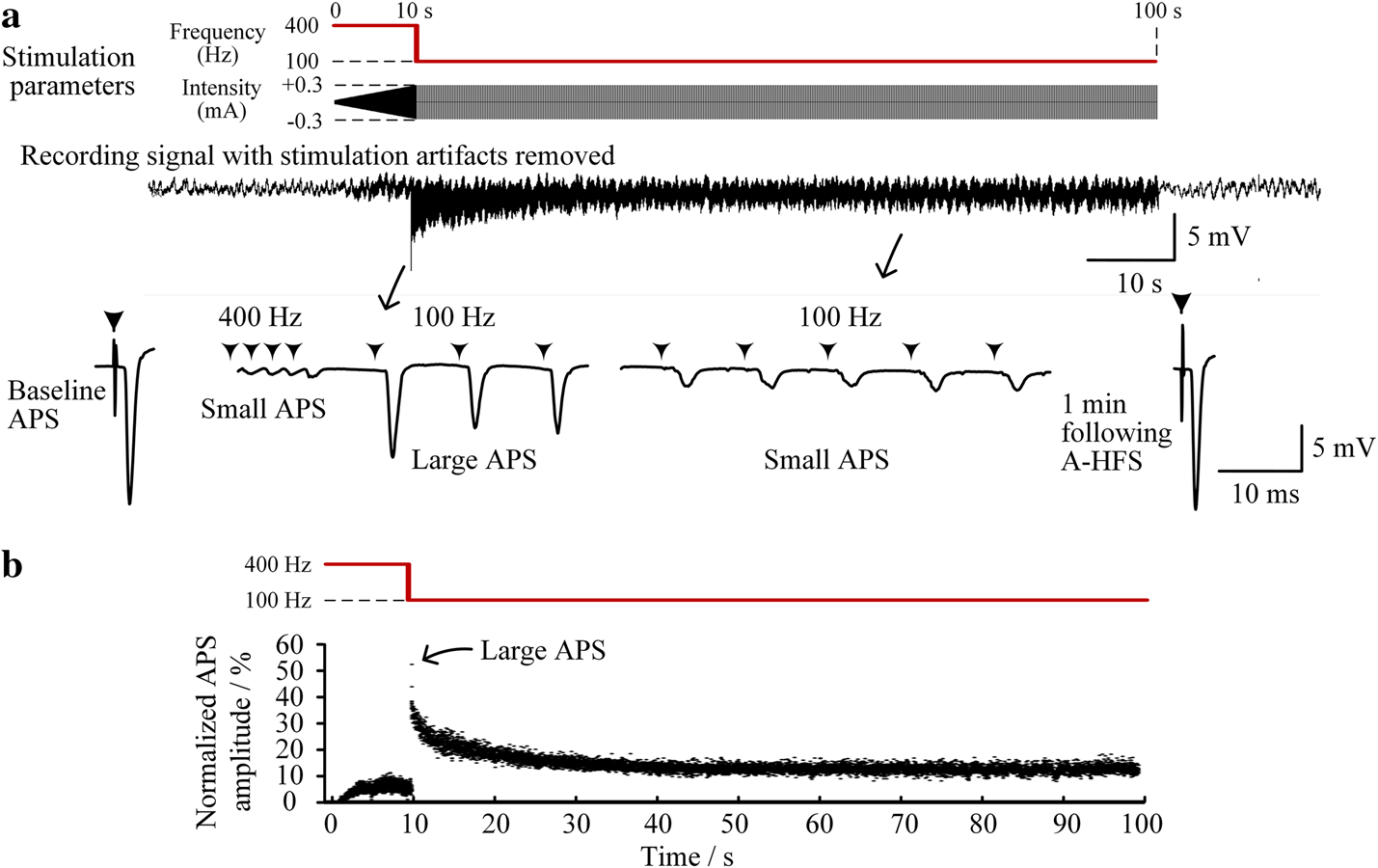
Neuronal responses to a time-varying stimulation paradigm. **a** A-HFS with a ramped-intensity and 400 Hz frequency suppressed the initial APS events, but large APS reappeared when the pulse frequency dropped directly from 400 to 100 Hz. *Top*: schematic diagrams of the changes of pulse frequency and intensity; *middle*: recording signal with stimulation artifacts removed; *bottom*: expanded plots of APS waveforms. **b** Scatter diagrams of the normalized amplitudes of APS evoked by each pulse during the 100-s stimulation

Utilizing a ramped-intensity from small intensity (one tenth of desired intensity, e.g., 0.03 mA) together with a high frequency 400 Hz at the initial 10 s of A-HFS, large initial APS disappeared (Fig. 6a). Even when the intensity increased to the desired intensity (e.g., 0.3 mA), the amplitudes of evoked-APS remained small (see the APS amplitudes in the first 10 s in Fig. 6b), similar to the values in the steady-phase of 400 Hz A-HFS with a constant 0.3 mA intensity (Fig. 5c).

**Fig. 7:**
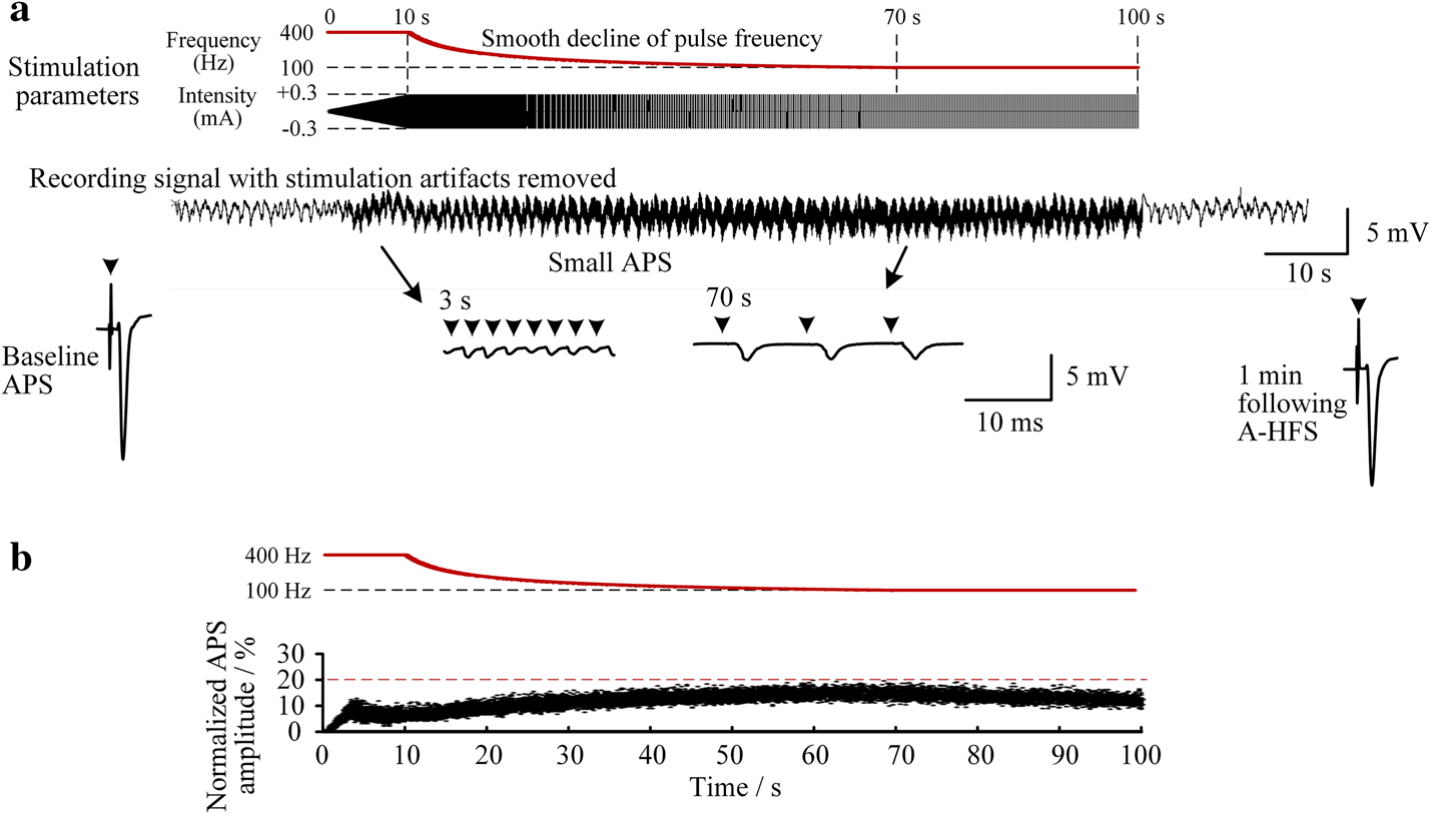
Neuronal responses to another time-varying stimulation paradigm. **a** Similar to the paradigm in Fig. 6 but decreasing the frequency smoothly and gradually to 100 Hz after the initial 10 s stimulation, no large APS appeared. **b** Scatter diagrams of the normalized amplitudes of APS evoked by each pulse during the 100-s stimulation

Although previous DBS investigations have shown that stable efficacy may remain up to a higher frequency 2000 Hz [21], a stimulation frequency of 400 Hz is higher than commonly-used DBS frequencies around 100 Hz and would consume more electrical power [11, 22]. Therefore, the frequency was decreased from 400 to 100 Hz following the initial 10 s stimulation. Nevertheless, large APS events reappeared upon the sudden drop of frequency. The neuronal response re-experienced a transient phase of large APS before decreasing to a steady phase of small APS (Fig. 6a, b). If the frequency decreased gradually from 400 to 100 Hz by a stimulation paradigm of attenuating frequency, the APS amplitudes would remain at a low level below 20% of the control (i.e., the baseline APS) without any large APS throughout the A-HFS (Fig. 7). Statistical data from six rat experiments showed that the mean APS amplitude (0.62 ± 0.31 mV) during a smooth-decline transition of frequency was significantly smaller than the mean APS amplitude (6.1 ± 2.1 mV) during a sudden drop of frequency (*P* < 0.01, *t*-test, *n* = 6). The results indicated that HFS with attenuating frequency could avoid generating large APS events during frequency transitions.

In all experiments, the APS evoked by a single pulse at one minute following the end of A-HFS was similar to the baseline APS before A-HFS (Figs. 5, 6, 7), indicating that the stimulation-induced changes of neuronal responses were reversible.

## Discussion

In this study, we designed a stimulation system to generate biphasic pulse sequences with time-varying stimulation parameters. Verification tests showed satisfactory functionality of the system. In addition, the results of validation experiments on rat hippocampus in vivo showed that time-varying stimulations could modulate neuronal activity in patterns differing from regular stimulations with constant parameters. The advantages of the stimulation system and its potential applications are described below.

Firstly, the stimulation system permits incorporation of adjustable time-varying parameters of both intensity and frequency into a pulse sequence. This extends the functions of commercial stimulators that only allow delivery of stimulation sequences with constant frequency and constant intensity. In addition, the LabVIEW GUI panel provides a virtual instrument and allows users to set parameters on-line during experiments. The system software is flexible and extensible, and could be easily replicated in other laboratories. It may control many types of commonly-used analog stimulators or stimulus isolators to meet the users’ demands. However, for a laboratory with little engineering assist, establishing the system and using LabVIEW may not be so easy. The system presented in this paper allows programming of three sequential stimulation paradigms (time-varying intensity, time-varying frequency, and constant parameters). More stimulation paradigms with more flexible user-programming capabilities need to be added to improve the versatility of the stimulation system in our further work.

Secondly, the intensity transition is designed as a linear change of magnitudes while the frequency transition is designed as a linear change of pulse intervals instead of a linear change of frequency itself. This paradigm of frequency transition enables a hyperbolic attenuating of frequency with fast initial decline transitioning into slow decline to the asymptote of a constant frequency. The smooth transition could avoid the synchronous excitation of neuronal population induced by an abrupt frequency change (see Figs. 6 and 7).

Thirdly, the preliminary animal experiments here showed interesting results revealing the effects of time-varying stimulation on neuronal activity. The amplitude of population spikes reflects the amount of neurons that fire action potentials synchronously [16]. Small APS potentials persisted in prolonged stimulation (see Figs. 5, 6, 7) may represent asynchronous neuronal firing induced by HFS, which has been considered as an important mechanism underlying DBS [23, 24]. The generation of asynchronous firing could be caused by a de-synchronized effect of HFS through HFS-induced axonal conduction failures [9, 10, 25]. However, synchronous firing could appear by a sudden change of stimulation intensity from smaller to larger (e.g., upon every onset of stimulation [10, 18]) or by a sudden change of stimulation frequency from higher to lower (Fig. 6). Nevertheless, the time-varying paradigms designed here could eliminate the synchronous firing that might induce epileptiform activity during brain stimulation. The possible mechanisms might be also related with axonal block induced by HFS [25] and await further investigations that might lead to new insights into mechanisms of DBS. Additionally, neuronal responses to these stimulation paradigms in other brain structures and regions are to be investigated in more animal experiments in future to determine their modulation effects.

Lastly, stimulation paradigms with varying parameters have been explored to advance DBS applications. For example, temporally patterned pulses with irregular inter-pulse-intervals have been applied in DBS to investigate their effects on tremor suppression [26] and motor symptom alleviation in Parkinson’s disease [8, 27, 28], as well as in epilepsy control [23, 29] and central nervous system arousal [30]. However, to our knowledge, a design of stimulator generating pulse sequences with smooth changes of time-varying parameters has not been reported before. In the present study, we used new stimulation paradigms with smoothly changing intensity and frequency to avoid intense firing of neuronal populations especially appearing at the initial period of stimulations. These paradigms may be particularly useful for certain circumstances such as closed-loop, or adaptive stimulations that may require switching between on and off states or among different stimulation parameters frequently [31].

In conclusion, the LabVIEW-based stimulation system provides a useful tool for developing new stimulation paradigms with adjustable time-varying parameters. It can meet potential requirements of various DBS modes. Utilization of new modulation modes may improve the efficacy of current DBS therapy and extend the clinical applications of DBS for treating more brain disorders.

## Abbreviations

DBS: deep brain stimulation
HFS: high frequency stimulation
GUI: graphical user interfaces
DAQ: data acquisition
VI: virtual instrument
APS: antidromically-evoked population spikes
A-HFS: antidromic high frequency stimulation
RE: recording electrode
ASE: antidromic stimulation electrode
